# Beyond sequence: Structure-based machine learning

**DOI:** 10.1016/j.csbj.2022.12.039

**Published:** 2022-12-29

**Authors:** Janani Durairaj, Dick de Ridder, Aalt D.J. van Dijk

**Affiliations:** a Biozentrum, University of Basel, Basel, Switzerland; b Bioinformatics Group, Department of Plant Sciences, Wageningen University and Research, Wageningen, the Netherlands

**Keywords:** Protein structures, Machine learning, Deep learning

## Abstract

Recent breakthroughs in protein structure prediction demarcate the start of a new era in structural bioinformatics. Combined with various advances in experimental structure determination and the uninterrupted pace at which new structures are published, this promises an age in which protein structure information is as prevalent and ubiquitous as sequence. Machine learning in protein bioinformatics has been dominated by sequence-based methods, but this is now changing to make use of the deluge of rich structural information as input. Machine learning methods making use of structures are scattered across literature and cover a number of different applications and scopes; while some try to address questions and tasks within a single protein family, others aim to capture characteristics across all available proteins. In this review, we look at the variety of structure-based machine learning approaches, how structures can be used as input, and typical applications of these approaches in protein biology. We also discuss current challenges and opportunities in this all-important and increasingly popular field.

## Graphical Abstract

**Figure:**
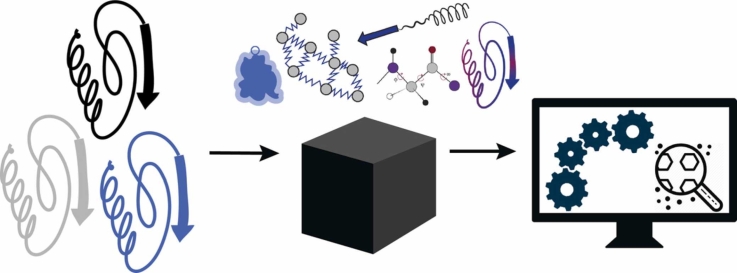

## 1 Introduction

Protein bioinformatics is a thriving and fast-growing field dealing with algorithms and data structures to explore, analyse and compare (groups of) proteins in order to better understand their various biological, physicochemical and molecular properties and functions. With the increase in protein sequence data obtained from large-scale high-throughput sequencing technology, machine learning (ML) has become a key methodology in protein bioinformatics. In protein structure prediction, be it secondary structure, backbone angles, contacts, folds, or full-atom structure, ML has become indispensable and forms the basis of a number of popular tools and algorithms. ML has also successfully been applied to predict protein function, protein-protein interactions, drug-target binding, enzyme substrate specificity, thermostability, catalytic rates, binding affinity, variant and mutant effects and more. ML is data-driven and attempts to identify patterns in existing data to predict properties of new, unseen data. Given ML’s requirement of large amounts of diverse data, the overwhelming majority of ML applications on proteins use sequences as input, some of which are powering different aspects of popular resources such as Ensembl [1], Pfam [2] and UniProt [3]. However, numerous protein families have divergent protein sequences yet share highly similar three-dimensional structures, topologies and folds, since structure tends to evolve slower than sequence [4]. Furthermore, protein tertiary structure typically provides a wealth of information not found in sequence - spatial topology, residue interactions, solvent accessibility, residue dynamics and electrostatics, and more.

Historically, structural biology depends primarily on experimental structure determination methods including X-ray crystallography, nuclear magnetic resonance (NMR), small-angle scattering, and cryo-electron microscopy (cryo-EM). The Protein Data Bank (PDB) [5], established in 1971, stores these experimentally determined structures and its size has been steadily increasing over the years. At the time of writing the PDB consists of 195,325 structures and grows by an average of 13,723 structures a year (calculated over 2017–2021). However, these numbers pale in comparison to the growing deluge of protein sequence data, with the UniProt protein database containing 226,771,949 sequence entries at the time of writing, over 771,752 more than the previous release with a release cycle of 8 weeks. This phenomenon is often referred to as the *sequence-structure knowledge gap* [6]. Fortunately, experimental approaches are not the only way to obtain structural information, and computational structure prediction techniques are fast closing this gap. A protein’s structure can be modelled from its sequence either using the experimental structures of one or more homologous proteins (template-based, comparative or homology modelling), or using *de novo* prediction techniques (template-free or *de novo* modelling). Given that homology modelling performs well when using templates with> 30% sequence identity to the protein of interest, accurate structural models can be obtained for over 60% of the genes in the top 12 most accessed genomes on UniProt [7], [8]. Template-free modelling, on the other hand, does not rely on global similarity to a known structure and hence can be applied to proteins with rarer folds. A recent breakthrough, the highly accurate deep-learning based AlphaFold2 model from DeepMind [9] trained on experimental structures to predict the structure for an input sequence, has allowed structural modelling to realise as high accuracy and resolution as the best experimentally resolved structures in many cases. In collaboration with EBI, DeepMind has released the AlphaFold Protein Structure Database [10], currently containing over 200 million structural models. This increases high quality structural coverage by an average of 25% compared to homology modelling across 11 proteomes [11], reaching over 76% for the human genome and reducing the fraction of the human “dark proteome” from 26% to 10% [12]. Thus, we can theoretically obtain high resolution protein structural information for a large number of available protein sequences. In addition, computationally predicted models can help better resolve experimental structures [13], [14], [15].

With these advances, we are at the brink of a structural revolution with millions of newly modelled structures at our disposal. Thus ML applications in protein bioinformatics, already shown to be very powerful in shedding light on biological problems, now have a wealth of structural information to exploit as input instead of, or along with, the typically used protein sequences. These sequence- and structure-based ML methods (hereafter referred to as “structure-based”) can greatly outperform purely sequence-based approaches, as demonstrated in studies where the same ML architecture is validated using only sequence and both sequence and structure information [16], [17], [18], [19], though sometimes data biases have prevented useful training of structure-based methods [20]. The past years have already seen movement in the direction of protein structure-based ML and its role is sure to increase drastically in future research. In this review, we describe the space of machine learning on protein structures in terms of the kinds of tasks that structures can help solve and the kinds of algorithms applicable to these tasks. We outline the various structural features and representations currently obtainable. Finally, we look at open problems and challenges, as well as promising opportunities in this exciting field.

## 2 Machine learning in the protein field

Machine learning (ML) is defined as “the study of computer algorithms that improve automatically through experience and by the use of data” [21]. Typically, these algorithms find patterns in datasets and link such patterns to specific outcomes or groupings. Deep learning (DL) is a sub-field of ML which uses artificial neural networks with multiple stacked layers of network connections enabling learning of increasingly complex information through huge amounts of data compared to the more “classical” ML approaches. In this work, we use ML to refer to both DL and classical ML.

Supervised ML attempts to predict a certain response by learning patterns from labelled data. In the case of classification, this response is the membership of the data point in a particular grouping or class. Regression, on the other hand, predicts a real-valued numeric outcome. Unsupervised ML attempts to find clusters or learn reduced representations from data without any labels. See [22] for an in-depth introduction to these topics.

ML has been used widely across biology for decades, with reviews outlining its usage in the fields of omics [23], synthetic biology [24], biomedicine [25], and drug discovery [26]. In the context of proteins, ML approaches, both supervised and unsupervised, can broadly be divided into **protein family** based and **protein universe** based techniques. These two categories differ in the kinds of prediction problems they are applied to, the kinds of algorithms used, and the kinds of representations used as input.

### 2.1 Protein family based ML

Protein family based ML is used to predict properties of the members of individual protein families or sub-families, usually consisting of hundreds to thousands of experimentally characterised training proteins. Some of the questions in protein family supervised ML include specificity prediction of substrates, intermediates, products, and inhibitors; state prediction in the context of engineering thermostability, binding affinity and activity; and prediction of the effects of mutations. In many cases, such as the immensely diverse lipocalins [27] and the fast-evolving enzyme families involved in specialised metabolism [28], the sequence diversity within a family make it impossible for sequence-based techniques to predict family properties. Even very similar sequences can have mutations in key structural regions resulting in completely different activities, which is easier to ascertain from structure than from sequence alone. In addition, insights from computational prediction methods which also use structure as input can better drive experimental studies due to the generally higher accuracy of structure-based prediction, and better enable exploration of the protein family space with structural stability and activity taken into account. We give examples of supervised ML tasks for some well known protein families below.

The superfamily of G protein-coupled receptors (GPCRs) is the largest family of targets for approved drugs in modern drug discovery, and hence also a popular target for ML approaches to drive exploration and understanding. GPCRs play an essential role in physiological processes such as vision, olfaction, neuronal signal transmission, cell differentiation, pain, muscle contraction, and hormone secretion [29]. Recent ML studies on GPCRs have started incorporating structural information to improve prediction performance, and to derive biological insight into the residues and mechanisms involved. As commonly used ML models for structure, interaction and interface prediction are trained on soluble proteins, specialised GPCR-specific oligomerization and interface predictors were developed [30], [31], able to handle their long transmembrane regions. Recent work even modified the existing AlphaFold2 algorithm to generate rarer GPCR conformations [32]. GPCRs often display high conformational flexibility and low thermostability, making their structural, biophysical, and biochemical characterisation in the laboratory challenging. Given that experimental identification of thermostabilizing mutations is very resource intensive and must be repeated for each individual receptor, computational prediction of GPCR mutant stability is a crucial task in this field [33]. Finally, GPCRs bind to a very diverse range of ligands and ML is used to identify biologically active ligands and binding inhibitors, estimating affinity and other binding properties, and probe ligand-specific binding mechanisms [34].

Another important class of drug targets are the kinases [35], with over 500,000 publications, 20,000 patents, inhibition assays for the majority of the human kinome and 115,000 kinase inhibitors covering 20% of the kinome [36]. With over 7000 structures solved covering 308 kinases across 8 groups and complexed with over 3000 unique ligands and inhibitors, structure-based ML approaches are widely used for addressing challenges within this superfamily. These include methods to predict inhibition [37] and binding affinity [38] in specific kinase families. Another common kinase challenge is predicting conformational change between the so-called active and inactive conformations [39], [40]. For drug targets, predicting the effects of mutation of a single protein could also be considered a protein family ML task, as the inputs are still proteins sharing the same structural fold with key differences caused by changes in the sequence. PremPLI [41] uses features from modelled protein-ligand complexes to predict the effect of mutation on binding affinity to a number of inhibitors for a kinase cancer target.

In the field of natural products and specialised metabolism in plants, bacteria, and fungi, ML has slowly been gaining popularity over more traditional approaches involving similarity search or analysis of a few, closely related proteins. ML has been used for successful prediction of substrate [42], [43] and product [44] specificity in various natural product enzyme families. In 2013, a structure-informed approach was used to engineer highly thermostable cytochrome p450s [19].

Though computationally predicted structures are shown to be highly accurate at the backbone level, tasks such as the ones described above which involve small molecule binding may need further family-specific processing and ML-based approaches to harness the structural information specifically related to ligand interaction. For example, [45] show that AlphaFold-predicted GPCR structures differ in crucial features such as domain assembly, ligand-binding pockets, and interface conformation, thus impeding their direct use in functional studies.

Unsupervised ML in the protein family space hosts a new sub-field of structural bioinformatics, dubbed “comparative structuromics" by Mohammed AlQuraishi. This is concerned with tools, algorithms, and techniques to compare and contrast assorted datasets of protein structures to answer a variety of biological questions - the evolutionary relationships between structural orthologs, interaction networks and how they are affected by structural changes, folding and changes within different cellular contexts and organisms, and how structure and folding are coupled with different functional characteristics. Zebra3D [46] is an example of such a technique. It provides a systematic analysis of 3D protein structure alignments combined with the identification of subfamily-specific regions using unsupervised ML clustering algorithms - these regions represent patterns of local 3D structure similar within subfamilies, but differing between them, thus likely to be associated with functional diversity and function-related conformational plasticity. The work of de Lima *et al.* [47] is another example of unsupervised protein family ML concerned with the detection of subfamilies and simultaneous identification of differentiating residues. Clustering and dimensionality reduction techniques have been used to describe the conformational landscape of proteins and identify binding-induced conformational change [48], [49].

a small number of data points. A wide range of algorithms are at our disposal for these tasks, including but not limited to k-nearest neighbours algorithms (k-NNs) [50], support vector machines (SVMs) [51], Gaussian processes [52], and ensemble methods such as Random Forests [53] and gradient boosting trees [54]. In addition, many approaches in this field aim to interpret prediction results to derive insights about underlying mechanisms and residues which may be important for function. Such predictions and insights obtained from protein family ML are often used to drive experimental research to explore and characterise novel, interesting or relevant proteins.

### 2.2 Protein universe based ML

The larger-scale protein universe based ML typically uses tens of thousands of proteins from diverse superfamilies to learn global properties of proteins, such as secondary and tertiary structure and folding, interactions, disorder, broad function classes etc. DL is a common choice for such problems, as it is known to drastically outperform other techniques in the presence of large amounts of data. In fact, protein structure prediction is in itself a protein universe task in which the use of DL has in many cases eclipsed other ML or statistical methods. This is true for prediction of secondary structure, solvent accessibility [55], backbone torsion angles [56], [57], residue-residue contacts or distance matrices from co-evolution [58], [59], [60], [61], [62], and in *de novo* all atom structure modelling. In fact, all the top-performing Critical Assessment of Structure Prediction (CASP13 [63], CASP14 [64]) methods for *de novo* modelling rely on deep convolutional neural networks for predicting residue contacts or distances, predicting backbone torsion angles and/or ranking the final models. For recent reviews on the underlying techniques used, including those in AlphaFold2 and related approaches, see [65], [66].

With the availability of protein structures, a number of additional tasks can make use of structure-based ML instead of sequence. These are listed in Table 1, grouped by the kinds of inputs used. Recent examples as well as common datasets used to validate and benchmark novel algorithms created for each task are also listed.

**Table 1:** Supervised protein universe tasks, inputs and examples.

Prediction of	Input	Examples	Datasets
Protein function	Protein	[67], [68]	SIFTS [69]
Mutant stability	Protein + Mutation	[70], [71], [72], [73]	ProThermDB [74], ATOM3D [75]
Cavity and pocket	Protein, Residue	[76], [77]	TOUGH-C1 [77], SOIPPA [78]
Model quality	Protein, Residue	[79], [80], [81], [82]	CASP [83]
PPI-Interface	Residue	[84], [85], [86], [87], [88], [89]	ProtCID [90], Docking benchmark v5 [91], DockGround [92], DIPS-Plus [93]
Ligand binding site	Residue	[94], [95], [96]	sc-PDB [97], COACH420 [98], HOLO4K [99]
Intrinsic disorder	Residue	[100], [101], [11]	DIBS [102], DisProt [103]
Interaction	Protein-protein complex, Protein + Protein	[104], [105], [106]	DIP [107], STRING [108], HPRD [109], BioGRID [110], HPIDB [111]
Protein binding affinity	Protein-protein complex, Protein + Protein	[112], [113], [114]	Affinity benchmark [91], SKEMPI2 [115]
Ligand screening and binding affinity	Protein-ligand complex, Protein + Ligand	[116], [117], [118], [119], [38], [120], [121], [122], [79], [123], [124]	PDBBind [125], Binding MOAD [126], DUD-E [127]

The Input column describes the typical form of input given to the algorithms used. Multiple input format possibilities are comma-separated. All inputs refer to the structural context, i.e. “Protein” refers to the 3D protein structure, “Residue” to aspects associated with each individual residue - its physicochemical, electrostatic, geometric properties etc. (similarly for “Mutation”), “Ligand” to the 2D and/or 3D structure of a small molecule ligand.

In the 2020 CASP14 competition, the breakthrough results of AlphaFold2 prompted a press release declaring the protein structure problem for single protein chains solved [64]. This emphasis on “single protein chains” revealed the new frontier for structural bioinformatics - complex structures are yet to be successfully predicted at the same breakthrough levels. Thus the related yet distinct tasks of predicting whether two proteins interact, and predicting the interface of two interacting proteins are common protein universe problems with a number of solutions, based on docking [104], [87], templates [105], end-to-end learning [84] and, most recently, protein complex prediction approaches building upon AlphaFold2 [129], [128], [130]. The latter generation combines the AlphaFold2 DL architecture with a modified paired MSA generation approach which encapsulates co-evolutionary information across the subunits of the desired complex. This yielded success rates for complex prediction up to double that of previous template-based and docking methods, marking significant progress in the field. However, these success rates are still only around 50% and vary drastically across species, protein families, types of complexes, and stoichiometries considered [129], [131]. Similarly, the popular *de novo* protein structure prediction algorithm RoseTTAFold, has been extended to the prediction of nucleic acid and protein-nucleic acid complexes [132], though again only around half of the tested complexes could be successfully modelled.

Structure-based drug discovery also hosts some significant applications of protein universe ML [133], starting from the computational modelling of putative receptor targets. Subsequently, binding sites in the target structure and putative drug candidates are identified using cavity/pocket prediction techniques [76], prediction of “druggable” regions, and protein-ligand binding site [134] prediction. This is typically followed by molecular docking to evaluate protein-ligand interaction and affinity between the target and a variety of drug candidates. In the case of unknown target proteins or to identify off-target binding candidates, reverse/inverse docking [135], [136], [137], [138] is used to create embeddings of drugs and search across protein structure databases for good docking solutions. In these contexts, ML approaches are used to improve scoring functions of binding affinity and plausible docking poses [121], [116], [81], [138], [139]. Indeed, [140] show that computationally predicted structures perform on par with experimental structures at reverse docking tasks - although the docking and scoring methods themselves could use major improvements to further drug discovery and design.

Predicting the effects of variants and mutations, especially those involved in diseases, is another common task. Sen *et al.* [141] took advantage of the latest *de novo* structure prediction techniques to model human disease-associated proteins, many of which do not have existing structures or even close homologues. Afterwards, they compared disease-associated mutations to ligand binding sites, protein-protein interfaces and conserved regions predicted from the models, in order to provide some rationale for most of the mutations. However, the current DL-based structure predictors are not yet able to successfully predict mutations in protein structures as their training procedure is designed to be robust to small changes in sequence. This has been practically demonstrated in studies aiming to predict stability effects of mutations using predicted structures [142], [143], and it indicates an under-explored area of structure prediction.

Approaches building upon AlphaFold2 and its underlying architectures have been used successfully in design tasks [144], [145], [146], [147], indicating that the AlphaFold2 breakthrough may also cause a leap in protein design prediction. The process of constructing idealised folds during protein design can reveal new information about the physical and structural constraints that dictate which conformations a protein can adopt [148], [149]. Such insights could be of vital importance to solving fundamental biological questions behind the evolution of proteins, as well as for further improvement of protein engineering and design [150]. See [151] for a recent review of DL approaches in the protein design field.

Instrinsically disordered proteins (IDPs) lack a fixed or ordered three-dimensional structure. This widespread phenomenon, thought to occur in over 33% of eukaryotic proteins, has been linked with allosteric regulation, enzyme catalysis, and a variety of diseases [152]. While structure-based prediction of intrinsic disorder may seem contradictory, energy scores obtained from existing structures [100] as well as residue-level computational modelling scores [101], [11] contain information correlating with disorder and are effective for prediction. Structure-based ML has also been used to sample the very diverse conformational ensembles of IDPs [153].

Unsupervised techniques in the protein universe support tasks such as structure query and retrieval, clustering for motif and hotspot discovery, and structure-based fold annotation. For the former task, an array of fast techniques that allows near-instant retrieval of structures matching an input structure [154], [155], [156], [157], [158]. Recent approaches for structure-based clustering allow pinpointing novel or rare folds [11], [159], as well as residues and structural regions associated with function [160]. Another common task is the generation of fixed-dimensional unsupervised embeddings which capture global and local protein characteristics. These can be used in downstream ML algorithms, as discussed in the next section.

## 3 Computational representations of protein structures

**Fig. 1:**
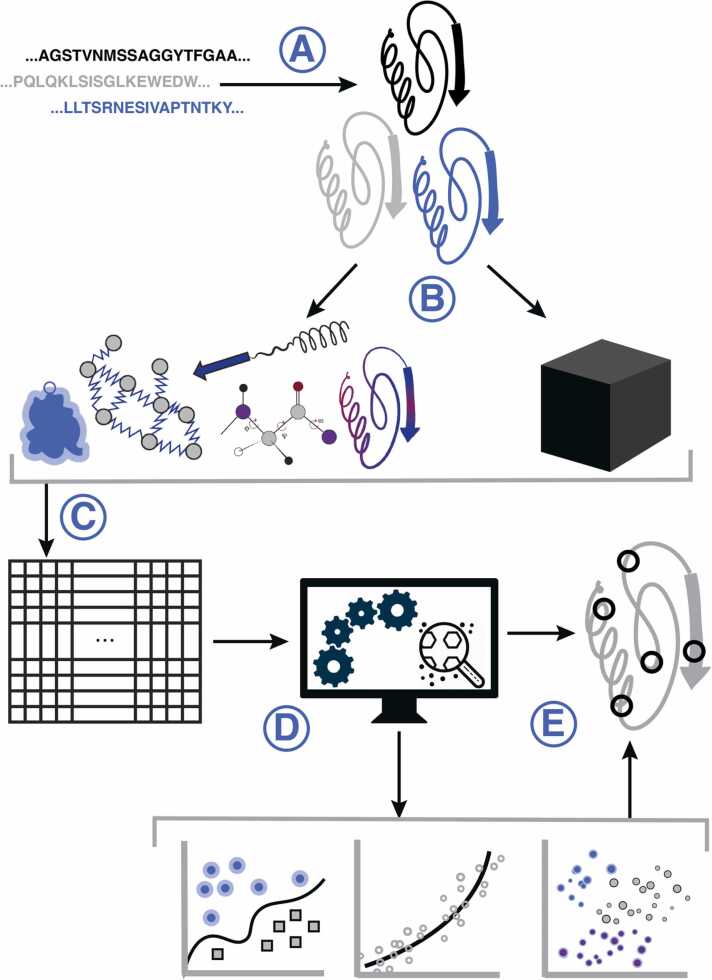
Common steps in structure-based machine learning. **A)** Starting from a set of protein sequences, structural models can either be retrieved from the PDB or constructed using computational approaches. **B)** A number of different feature extraction, feature engineering, or pre-trained embedding approaches can then be used **C)** to extract a matrix representation of the input, with the rows as data points and columns representing features or embedding values. **D)** This matrix forms the input for ML models resulting in predictions of classes, regression values, or unsupervised clustering and dimensionality reduction. **E)** Prediction results, combined with the trained model, can be used to inspect and interpret regions of the protein structure relevant for the task at hand.

Protein structures contain interconnected high-dimensional information about the amino acids involved, their positions and relative orientations, and the varying physicochemical and electrostatic effects they have on each other. Fig. 1 shows an overview of the most common steps taken in structure-based ML. Once a set of structures with or without associated labels has been collected (Fig. 1A), the next step typically consists of choosing a format to represent this information that can be understood by computers (Fig. 1C). One way to do this is by explicitly extracting a set of attributes or features from proteins to create a tabular *feature matrix*. Another approach is to generate reduced fixed-dimensional protein representations, referred to as *embeddings*. Both these approaches (Fig. 1B) are followed by the use of ML algorithms that take the feature matrix or embedding as input and return various results (Fig. 1D) and insights (Fig. 1E) for user interpretation.

A number of studies have demonstrated that high-confidence predicted structural models (both homology-based and DL-based) have predictive power and can even perform as well as experimental structures on specific tasks [161], [33], [16], [11]. However, this is unlikely to be a general statement as it is highly dependent on both the types of proteins and the task at hand. For example, membrane proteins, intrinsically disordered proteins, and proteins with high conformational flexibility would still benefit from experimental structures solved in different conditions to increase the diversity of structures available and thus our knowledge of them. In addition, side-chain modelling accuracy, crucial for tasks involving side-chain interactions, tends to lag behind main chain accuracy. Finally, in a significant number of cases, AlphaFold2 and related approaches do not produce high-confidence structures. It was recently shown that while residues predicted by AlphaFold2 with high confidence (> 90 plDDT) have a very low prediction error (median 0.6 Å), this quickly increases to over 3 Å error for low confidence residues (< 70 plDDT) [162]. For such cases with only low confidence structure information present, we may still have to fall back on sequence-based approaches or utilise embedding techniques as described in Section 3.2.

### 3.1 Generating structure feature matrices

Broadly, protein structures are compared at the residue level, where features are extracted from each individual residue in the structure, or at a structural environment level, where features are extracted from well-defined portions of the structure (or the entire structure) containing relevant and localised properties. The former approach is commonly used in structurally conserved protein family ML tasks involving the entire protein, and the latter is used for more divergent proteins or for more specific tasks involving the corresponding structural environments. Both approaches use a range of techniques to align or arrange the extracted features into the fixed dimensional feature matrix format.

#### 3.1.1 Residue level

Many different features can be extracted from each residue in a protein structure using a plethora of computational tools, as listed in Table 2.

**Table 2:** Structural features and tools used to extract them. Apart from DISPORED, all tools use protein structures as input.

Residue feature	Tools
Accessible surface area	NACCESS [163], PSAIA [164], FreeSASA [165], DSSP [166], ProtDCal [167]
Half sphere exposure	BioPython (Bio.PDB.HSExposure) [168]
Residue depth	MSMS [169], PSAIA [164]
Hydrogen bonding patterns	DSSP [166]
Bond angles	DSSP [166], MDAnalysis [170]
Secondary structure	DSSP [166]
Energy	FoldX [171], Rosetta [172]
Electrostatics	APBS [173]
Disorder	DISOPRED [174]
Residue flexibility and stiffness	ProDy [175], MechStiff [176]
Perturbation response	PRS [177]
Thermodynamics	ProtDCal [167]

When the proteins under consideration are evolutionarily closely related, multiple protein alignment is commonly used to generate the input feature matrix. While sequence alignment has generally been much more popular than structure alignment, the existence of protein families which share the same structural fold despite having little sequence similarity necessitates the use of structure-based alignment methods. This has driven the development of fast multiple structure aligners capable of scaling to the numbers of proteins required to train ML algorithms [178], [179], [180].

An alternative to the tabular format is a (dis)similarity matrix, often used as input to kernel-based methods such as SVMs or in unsupervised dimensionality reduction. For instance, de Lima *et al.* [47] calculate protein-protein similarity by combining similarities calculated from, among other features, structural alignment, alignment-free structural comparisons, putative active sites, and instability indices.

#### 3.1.2 Structural environment level

**Fig. 2:**
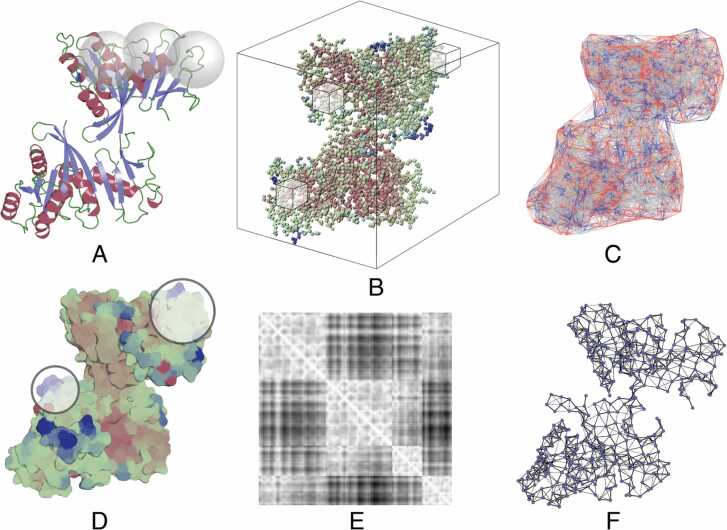
Different approaches for computational representation of a protein structure which go beyond features of individual residues. For **A-D** features or representations calculated across individual blocks (respectively: spheres, grids, polyhedra, surface patches) are used as input to ML, while for **E-F**, the entire matrix or graph is often used in methods specifically designed for these kinds of inputs. **A** Overlapping spheres **B** 3D voxel grids **C** Geometric tesselations **D** Molecular surface representations **E** Distance/contact maps **F** Graph representations.

Fig. 2 depicts some structural environments commonly used in computational representations. For tasks such as hotspot prediction or interface residue prediction, each input data point could be a single residue. In such situations, including aggregate features with weighted neighbour averages over the spatial nearest neighbouring residues, as shown in Fig. 2A, often improves the discriminatory power of predictors [181]. Some environment representations were borne out of ease of adaption of approaches from other fields to protein structures - for example, viewing the three-dimensional coordinates of atoms in a structure as a 3D image grid (Fig. 2B) allows the application of voxelization followed by the use of 3D convolutional neural networks often applied in the field of computer vision. Whereas in the case of images the red, green and blue values are often encoded as different channels, for proteins these channels have been used to encode different atom types [77], [95]. Another approach that can also take into account atomic density and radii is the use of geometric tessellations to define a set of polyhedra around atoms or residues in a structure [182], [183], [184], [185] (Fig. 2C).

Representations of the molecular surface (Fig. 2D) are useful for tasks related to protein interactions and protein-solvent interactions. For example, MaSIF [86] depicts the surface as a series of overlapping radial patches with associated geometric features such as shape index and distance-dependent curvature, as well as chemical features such as hydropathy index, continuum electrostatics and the location of free electrons and proton donors. A geometric deep neural network is applied to these input features to spatially localise features and optimise them towards particular tasks. Other approaches have used 3D Zernike or similar descriptors of surfaces which are invariant to rotation, thus allowing structures and surfaces of different proteins to be compared [186], [187], [188]. In fact, one of the main problems to solve when representing entire protein structures is this rotational and translational invariance. Fig. 2E depicts one way to address this, namely by using a 2D residue-residue distance or contact map [189], [190]. Another approach gaining popularity is the representation of a protein structure as a graph (Fig. 2F) with rotation and translation invariant properties attached to the nodes and/or edges [191], [192], [193], [17], [194]. These graphs form the ideal input for geometric deep learning approaches and have the capacity to encode most of the information contained in the protein structure [195], [196].

Proteins often interact with other molecules - other proteins, peptides, nucleic acids and small molecule ligands - so computational representations of these binding regions or interfaces are necessary for a number of tasks. Graph [197], [122], [198] and voxel-based [79], [116], [199] approaches can be used on experimentally solved or computationally docked protein-ligand complexes, usually by zooming in to the ligand binding pocket. In addition, there are specialised approaches to take into account explicit protein-ligand interactions within the ligand binding pocket in a complex [200], [124]; see [201] for more examples of protein-ligand feature representations. In cases where data about the complex is absent but unbound structures are present, some approaches concatenate features of the individual entities as their representation [117], [120], [119].

### 3.2 Learning protein embeddings

A complementary approach to generate the tabular input required for ML is by using end-to-end or pre-trained embedding algorithms. These typically make use of unsupervised DL methods trained on a large dataset of proteins to produce a series of values representing a given protein in a fixed high-dimensional space, often without the need for explicitly handcrafted features. Due to the training process, these values place similar proteins closer together in this space thus capturing overall protein variation and relationships between individual proteins. For example, recent global sequence embeddings have been shown to capture amino acid characteristics and other physiological properties of proteins as a whole [202], [203], [204], [205]. These have recently been extended to include structural information as well [206], [207]. Unlike protein family ML, alignment is generally not an option in such techniques since most proteins used for training are evolutionarily remote, thus most described embedding techniques depend on learning alignment-free patterns across diverse proteins or on generating on-the-fly alignments of sub-groups of data during the learning process.

End-to-end learning is popular in this area, covering techniques which start from the raw protein structure with minimal processing and automatically extract features based on optimising prediction accuracy in a given end task - thus the intermediate feature representations or embeddings learned are more applicable to the task at hand and can be retrained to adapt better to different tasks. ContactLib-ATT [208] applies this concept to predict the SCOP (Structural Classification Of Proteins) classification of an input structure, using attention-based learning [209] on vectors of hydrogen bond properties extracted from the structure. SASNet [84] is an example of such an approach applied to interface prediction. Local atomic environments of each surface residue are voxelized and a 3D convolutional neural network is applied to the resulting grids of each pair of residues to learn their interaction propensity. Interestingly, this method was trained based only on residues within bound structures of interacting partners and yet performs exceedingly well also on unbound counterparts, indicating that complex features beyond simple shape complementarity can be learned in this end-to-end fashion. dMaSIF [210], the successor to MaSIF (mentioned above), performs end-to-end learning of molecular surface representations directly from 3D point cloud data, optimised to each prediction task. Removing the reliance on handcrafted features improved the running time of dMaSIF by many orders of magnitude compared to MaSIF while maintaining and even improving accuracy. Recent DL approaches use the concept of “equivariance” (i.e rotation and translation of coordinates does not affect the learning process) in sequence, graph-based, and diffusion architectures for end-to-end predictive and generative learning [211], [212], [213], [213].

GeoPPI [113] is an unsupervised approach that operates on the graph of a protein complex and uses a message passing neural network to reconstruct the structure of a perturbed complex, i.e one in which a random residue is modified. This enables learning of intrinsic binding interactions, optimal for the prediction of protein-protein binding affinity. An advantage of such “self"-supervised approaches is that they are not specific to a single task while still encoding more global protein context; i.e GeoPPI embeddings could easily be used as input for any prediction task. This kind of repurposing of unsupervised or pretrained embeddings is quite popular in the sequence world [214], [215], and likely the same will hold through for structure-based ML in the future. Pretrained embeddings can also be used in a transfer learning context, where they are further fine-tuned to a more specific case of a general protein problem, such as the prediction of antibody-antigen interfaces from an embedding trained across all protein-protein interfaces [17].

Another interesting and relevant approach is structure-guided sequence embeddings [216], [203], [217] - these make use of structural information only in the training stage while the input to the embedding algorithm from the perspective of the end user is just the sequence. This provides a compromise between the use of structure data, which may be computationally expensive to produce, and more easily accessible sequence data while still making use of implicit structural information. Some recent work [218], [194] has even made use of the intermediate representations generated by AlphaFold2 during the structure prediction process instead of, or along with, the predicted structure itself - these representations contain information about homologous sequences and structures, especially useful for predicting the effects of mutations or ligand binding, most of which is lost on generation of the final structure.

## 4 Challenges and future directions

Despite rapid progress in the direction of structure-based ML, there are challenges to address before it can become as ubiquitously used as sequence-based ML. Just as there exists a wide variety of tools for answering questions from a sequence perspective, there need to be tools in structural bioinformatics that are as easy to use, as intuitive to interpret, as optimised, and as feature-rich.

### 4.1 Structure-based approaches are computationally expensive

The universal and widespread use of protein sequence data, combined with its one-dimensional nature, has resulted in a diverse landscape of highly optimised sequence-based tools and algorithms. Many of these, including clustering algorithms, aligners, feature extractors etc., scale to hundreds of thousands of sequences with ease. This cannot be said for structure-based approaches yet, both due to their relative newness and to structural data being much more complex than sequence data.

Often this resource intensiveness starts from the very first step - i.e. generating structural models. Template-based or homology modelling approaches take a matter of minutes to hours for generating a single model, often exacerbated by the need to infer multiple models for better robustness and expensive additions such as loop modelling for special cases. Recent template-free methods such as AlphaFold2 and RosettaFold run in minutes, though scaling very poorly with the number of residues, and require GPUs and high amounts of memory and disk space. Memory and space requirements for both are somewhat alleviated by the presence of servers such as SWISS-MODEL [219] for template-based modelling and the recently released ColabFold [220] for template-free modelling, both of which allow running these resource intensive modelling steps on shared external servers. In addition, the growth of the AlphaFold protein structure database [9] will eventually reduce the need for remodelling from scratch for a large number of sequenced proteins. Mutants, designed and novel proteins will still need computational modelling however, indicating that speeding up the modelling process is still a relevant problem in the field. Recent approaches that use protein language model embeddings as input instead of calculating time-intensive multiple sequence alignments (MSAs) provide a step in this direction [221]. With the growth of exascale computing resources, modelling structural dynamics via molecular simulations is increasingly accessible, though there is a long way to go for this to become commonplace.

Once a dataset of structures is gathered or generated, the next steps often involve structural comparison and feature extraction. Alignment-free structural comparison techniques are relatively fast already, but structural aligners that scale to the sizes of datasets required for ML have only recently started to appear. These are still a far cry from the highly optimised sequence aligners, but many of these optimisation techniques can be transferred to structure-based approaches and represent a logical next step as ML on structures grows in popularity. Extraction of many of the features detailed in Table 2 is time consuming as well. While some improvements can be made with parallelisation and making better use of modern hardware, this is unlikely to scale to hundreds of thousands of proteins in a similar timescale as sequence feature extraction.

### 4.2 End-to-end learning on structures

End-to-end learning, where a DL model learns a mathematical function to map an input to a complex output [222], with minimal handcrafting of intermediate features and tasks, was seen to be highly successful for the extremely complex task of mapping an input sequence to a 3D structure [66]. This has been followed by a boom in end-to-end learning approaches on proteins sequences for function prediction, as well as on protein structures for generating designed protein sequences. See [223] for a recent review.

End-to-end learning is becoming popular for a number of tasks as large models trained once on huge datasets of structures can then be reused for smaller sets of proteins and adapted to similar tasks with much less resource consumption and, at the same time, a great increase in performance for even sparse amounts of data [224], [16], [213], [225], [212]. In addition, these approaches can learn to make use of relevant intermediate information from proteins that may not be required or prioritised for the structure prediction task but are crucial for other downstream tasks - for example, residue masking in the AlphaFold2 learning procedure increases its robustness and improves overall structure prediction but makes it impossible to predict the structural changes caused by mutations, while much of this information is still present in the intermediate representations and useful for mutant effect prediction [218].

However, these learners do need huge initial training sets of diverse data and careful architecture engineering to avoid overfitting as well as large amounts of computational resources for training and inference. In addition, results from such approaches are difficult to interpret in terms of which kinds of protein properties are being used to make certain decisions, which is a useful property of more handcrafted ML techniques to hypothesise about the underlying biology.

### 4.3 Dynamic representations of structure

Since proteins are inherently dynamic in nature, their true “structure” is much more than the rigid three-dimensional coordinates which serve as the basis for many of the approaches detailed in the previous sections. Instead, a protein is an ensemble of possible conformations, with some areas displaying more flexibility than others. This is further influenced by the constant interaction of proteins with the surrounding solvent, small molecules, nucleic acids, peptides and of course other proteins, all of which drive conformational changes within the protein. Protein biological activity often involves adopting specific conformations, contributions from local fluctuations, and even large-scale structural transitions between different conformations. In fact, the old paradigm that sequence encodes structure, and structure determines function can now be rephrased as sequence encodes structure, structure determines dynamics, and dynamics encodes function [226].

Protein flexibility and conformational diversity can be modelled in multiple ways. One of the most common approaches is using molecular dynamics (MD) simulations, which calculates the force exerted on each atom by all other atoms as a function of time using a molecular mechanics force field [227]. However, MD simulations, which are already computationally extremely expensive, do not address covalent bond formation or breakage, both crucial in a number of enzyme families. This sometimes leads to the need for the even more expensive and challenging set up of Quantum mechanics/molecular mechanics (QM/MM) simulations [228]. Coarse-grained modelling with Monte Carlo simulations (CG-MC) and elastic network models (ENM, a.k.a normal mode analysis) both provide simplified protein representations that still allow for understanding some aspects of protein flexibility while greatly reducing computational time [226], [229]. structures resolved by cryo-EM, a fast-growing number.

Together, these computational techniques can provide information about globular protein flexibility and mutations [230], [231], large-scale structural transitions (e.g.from active to inactive conformations) [232], [233], [234], [235], and conformations involved in the formation of protein complexes [236]. They have also been used to assess and refine 3D models [237], [238], [239], improve ligand positioning [240], [241], and to create receptor ensembles for ensemble docking [242], [243]. The faster and cruder CG-MC and ENM approaches can be combined with atomistic-level MD, providing efficient strategies and starting points for multiscale simulations of proteins and complexes [244]. While ML is becoming more prevalent in the MD and CG-MC fields, to construct force field models, model energy surfaces, and perform conformational sampling [245], [246], [247], future efforts will likely also utilise the flexibility information obtained from these techniques to use as input in ML-based predictors of protein function, with a few early examples already doing this in unsupervised [248], [249] and supervised settings [250], [251]. There is some evidence that this can improve over static structure-based prediction [252].

### 4.4 Probing underlying protein mechanisms

A major limitation of DL-based structure prediction techniques, where prediction acts merely as an alternative to an experimental technique, is that they do not immediately provide us with a deeper understanding of the processes behind the folding of proteins as this is not their aim [253]. In contrast, many approaches using structural data to predict protein properties, especially those in protein family ML, have tried to make more explicit use of the rich feature sources provided to extract mechanistic insights and interpret the residues, causes and processes involved behind specific predictions, as well as guide experimental design in the most relevant directions.

Interpretable ML is a crucial concept in bioinformatics, as often we are as interested in the how and why of a prediction as we are in the what. Thus an important next step in structure-based ML is to couple predictions with an understanding of protein biology in terms of folding, interaction, function, and the interplay between the three. From a protein universe perspective, interpretation becomes dependent on the model inspection techniques specific to DL approaches. While this is a nascent field, techniques such as integrated gradients, saliency and class activation maps exist for this purpose, though they are rarely used yet in structure-based ML tasks [254]. Large-scale unsupervised techniques exploring the protein structural space can also be helpful to pinpoint folds, pockets, and interfaces upon which evolutionary and function-specific analyses can be conducted and for which ML representations and techniques that lend well to linking of prediction to cause can be used. Most importantly, a tight coupling of computational prediction with experimental set up is required, creating a feedback loop that improves prediction and experimentally characterizes relevant functional space.

### 4.5 A unified approach to function

Biological function is only partly determined by an individual protein – its genomic and cellular contexts also play a big role. Each protein is determined by an underlying gene sequence, but the mapping from gene to protein is not so straightforward, complicated by the existence of alternatively spliced transcript variants [255], pre-protein sequences in need of further processing [256], and moonlighting pseudoenzymes [257]. In addition, post-translational modifications, the developmental stage of an organism’s life, their subcellular localisation and environment in the cell, and even the extra-cellular conditions all have an effect on protein expression and function [258]. More often than not, proteins also work in concert with a wide variety of other entities, ranging from metal ions and cofactors, water and other solvent molecules, small molecule ligands, peptides, nucleic acids, and other proteins.

One area of study focused on integrating these different contexts of proteins and their complex interactions is network biology. This field is crucial for the accurate modelling of biological systems, and given the influx of data from high-throughput interaction assays and large-scale multi-omics studies, a great target for ML and DL methods. The future holds an increasing number of opportunities for this combination of network biology and ML [259] – in understanding and fighting diseases by inspecting protein and gene interaction networks, in locating off-target effects of drugs and concocting valuable drug combination therapies based on chemical networks and multi-omics data from drug treatments [260], in understanding microbial interactions through metabolic networks, in finding biosynthetic gene clusters through gene neighbourhoods, transcriptomics, and expression profiling, and in designing synthetic gene circuits combining interconnected genes, promoters, and ribosome binding sites. Apart from a few examples [261], structural data has rarely been used in such large scale integrative approaches due to its scarcity and complexity. With the former being solved, the future holds promise in finding and using algorithms and approaches to link protein structures with all of their interlinked data in a unified approach to model function [262].

## 5 Conclusion

Protein structure is a central component to understanding biological processes, and thus a great addition to ML approaches in the protein bioinformatics field. In this review we described the space of structure-based ML in terms of the tasks it can be applied to, and the kinds of input representations and algorithms used with a number of examples demonstrating the powerful predictions that can be obtained. Mainly due to the recent breakthroughs in computational structure prediction, the field of structure-based ML is expanding very rapidly, with a high number of actively cited preprints in this review attesting to this. At the moment, sequence-based features, aligners, representations, and ML approaches still far outnumber structure-based ones and they are generally much faster as well. However, the power of structural information to improve computational prediction of protein biology is alluring, and the growth of structural databases, algorithms for alignment and representation, and increasing accessibility of relevant DL approaches and architectures will foster a new generation of protein bioinformatics in which structure will play a starring role.

## CRediT authorship contribution statement

**Janani Durairaj**: Conceptualization, Investigation, Visualization, Writing – original draft, Writing – review & editing. **Dick de Ridder**: Writing – review & editing, Supervision. **Aalt D.J. van Dijk**: Writing – review & editing, Supervision, Project administration.

## Conflicts of Interest

We have no conflicts of interest to disclose.

## References

[1] Zerbino D.R., Wilder S.P., Johnson N., Juettemann T., Flicek P.R. (2015). The ensembl regulatory build. Genome Biol.

[2] Bileschi M.L., Belanger D., Bryant D.H., Sanderson T., Carter B., Sculley D., Bateman A., DePristo M.A., Colwell L.J. (2022). Using deep learning to annotate the protein universe. Nat Biotechnol.

[3] Gane A., Bileschi, M.L., Dohan D., Speretta E., Héliou A., Meng-Papaxanthos L., Zellner H., Brevdo E., Parikh A., Orchard S. ProtNLM: model-based natural language protein annotation.

[4] IllergÅrd K., Ardell D.H., Elofsson A. (2009). Structure is three to ten times more conserved than sequence–a study of structural response in protein cores. Proteins Struct Funct Bioinform.

[5] Berman H.M., Westbrook J., Feng Z., Gilliland G., Bhat T.N., Weissig H., Shindyalov I.N., Bourne P.E. (2000). The protein data bank. Nucleic Acids Res.

[6] Schwede T. (2013). Protein modeling: what happened to the “protein structure gap”?. Structure.

[7] Somody J.C., MacKinnon S.S., Windemuth A. (2017). Structural coverage of the proteome for pharmaceutical applications. Drug Discov Today.

[8] Bienert S., Waterhouse A., de Beer T.A.P., Tauriello G., Studer G., Bordoli L., Schwede T. (2017). The SWISS-MODEL Repository–new features and functionality. Nucleic Acids Res.

[9] Jumper J., Evans R., Pritzel A., Green T., Figurnov M., Ronneberger O., Tunyasuvunakool K., Bates R., Žídek A., Potapenko A., Bridgland A., Meyer C., Kohl S.A.A., Ballard A.J., Cowie A., Romera-Paredes B., Nikolov S., Jain R., Adler J., Back T., Petersen S., Reiman D., Clancy E., Zielinski M., Steinegger M., Pacholska M., Berghammer T., Bodenstein S., Silver D., Vinyals O., Senior A.W., Kavukcuoglu K., Kohli P., Hassabis D. (2021). Highly accurate protein structure prediction with AlphaFold. Nature.

[10] Varadi M., Anyango S., Deshpande M., Nair S., Natassia C., Yordanova G., Yuan D., Stroe O., Wood G., Laydon A. (2022). AlphaFold Protein Structure Database: Massively expanding the structural coverage of protein-sequence space with high-accuracy models. Nucleic Acids Res.

[11] M. Akdel, D.E.V. Pires, E.P. Pardo, J. Jänes, A.O. Zalevsky, B. Mészáros, P. Bryant, L.L. Good, R.A. Laskowski, G. Pozzati, A. Shenoy, W. Zhu, P. Kundrotas, V.R. Serra, C.H.M. Rodrigues, A.S. Dunham, D. Burke, N. Borkakoti, S. Velankar, A. Frost, J. Basquin, K. Lindorff-Larsen, A. Bateman, A.V. Kajava, A. Valencia, S. Ovchinnikov, J. Durairaj, D.B. Ascher, J.M. Thornton, N.E. Davey, A. Stein, A. Elofsson, T.I. Croll, P. Beltrao, A structural biology community assessment of AlphaFold2 applications, Nat Struct Mol Biol 29(11) (2022) 1056–1067. 10.1038/s41594-022-00849-w. PMC9663297 36344848

[12] Porta-Pardo E., Ruiz-Serra V., Valentini S., Valencia A. (2022). The structural coverage of the human proteome before and after AlphaFold. PLoS Comput Biol.

[13] Pfab J., Phan N.M., Si D. (2021). Deeptracer for fast de novo cryo-em protein structure modeling and special studies on cov-related complexes. Proc Natl Acad Sci USA.

[14] Jin S., Miller M.D., Chen M., Schafer N.P., Lin X., Chen X., Phillips G.N., Wolynes P.G. (2020). Molecular-replacement phasing using predicted protein structures from awsem-suite. IUCrJ.

[15] Chai L., Zhu P., Chai J., Pang C., Andi B., McSweeney S., Shanklin J., Liu Q. (2021). Alphafold protein structure database for sequence-independent molecular replacement. Crystals.

[16] Abdin O., Nim S., Wen H., Kim P.M. (2022). PepNN: a deep attention model for the identification of peptide binding sites. Commun Biol.

[17] Pittala S., Bailey-Kellogg C. (2020). Learning context-aware structural representations to predict antigen and antibody binding interfaces. Bioinformatics.

[18] Liu R., Hu J. (2013). DNABind: a hybrid algorithm for structure-based prediction of DNA-binding residues by combining machine learning- and template-based approaches. Proteins Struct Funct Bioinform.

[19] Romero P.A., Krause A., Arnold F.H. (2013). Navigating the protein fitness landscape with Gaussian Processes. Proc Natl Acad Sci USA.

[20] Volkov M., Turk J.-A., Drizard N., Martin N., Hoffmann B., Gaston-Mathé Y., Rognan D. (2022). On the frustration to predict binding affinities from protein-ligand structures with deep neural networks. J Med Chem.

[21] Mitchell T.M. (1997).

[22] Greener J.G., Kandathil S.M., Moffat L., Jones D.T. (2022). A guide to machine learning for biologists. Nat Rev Mol Cell Biol.

[23] Li R., Li L., Xu Y., Yang J. (2022). Machine learning meets omics: applications and perspectives. Brief Bioinform.

[24] Sieow B.F.-L., De Sotto R., Seet Z.R.D., Hwang I.Y., Chang M.W., Selvarajoo K. (2023). Computational biology and machine learning for metabolic engineering and synthetic biology, methods in molecular biology.

[25] Wainberg M., Merico D., Delong A., Frey B.J. (2018). Deep learning in biomedicine. Nat Biotechnol.

[26] Vamathevan J., Clark D., Czodrowski P., Dunham I., Ferran E., Lee G., Li B., Madabhushi A., Shah P., Spitzer M., Zhao S. (2019). Applications of machine learning in drug discovery and development. Nat Rev Drug Discov.

[27] Flower D.R., North A.C.T., Sansom C.E. (2000). The lipocalin protein family: structural and sequence overview. Biochim Biophys Acta ((BBA)) Protein Struct Mol Enzymol.

[28] Durairaj J., DiGirolamo A., Bouwmeester H.J., de Ridder D., Beekwilder J., van Dijk A.D. (2019). An analysis of characterized plant sesquiterpene synthases. Phytochemistry.

[29] Böhme I., Beck-Sickinger A.G. (2009). Illuminating the life of GPCRs. Cell Commun Signal.

[30] Barreto C.A.V., Baptista S.J., Preto A.J., Matos-Filipe P., Mourão J., Melo R., Moreira I., Giraldo J., Ciruela F. (2020). Progress in molecular biology and translational science, Vol. 169 of oligomerization in health and disease: from enzymes to G protein-coupled receptors.

[31] Bordner A.J. (2009). Predicting protein-protein binding sites in membrane proteins. BMC Bioinform.

[32] L. Heo, M. Feig, Multi-state modeling of G-protein Coupled Receptors at experimental accuracy, bioRxiv Preprint (Nov. 2021). 10.1101/2021.11.26.470086. PMC9561049 35510704

[33] Popov P., Peng Y., Shen L., Stevens R.C., Cherezov V., Liu Z.-J., Katritch V. (2018). Computational design of thermostabilizing point mutations for G Protein-Coupled Receptors. eLife.

[34] Raschka S., Kaufman B. (2020). Machine learning and AI-based approaches for bioactive ligand discovery and GPCR-ligand recognition. Methods.

[35] Cohen P. (2002). Protein Kinases — the major drug targets of the twenty-first century?. Nat Rev Drug Discov.

[36] Laufer S., Bajorath J. (2014). New frontiers in kinases: second generation inhibitors. J Med Chem.

[37] Afanasyeva A., Nagao C., Mizuguchi K. (2020). Developing a kinase-specific target selection method using a structure-based machine learning approach. Adv Appl Bioinform Chem AABC.

[38] de Ávila M.B., Xavier M.M., Pintro V.O., de Azevedo W.F. (2017). Supervised machine learning techniques to predict binding affinity A study for Cyclin-Dependent Kinase 2. Biochem Biophys Res Commun.

[39] McSkimming D.I., Rasheed K., Kannan N. (2017). Classifying kinase conformations using a machine learning approach. BMC Bioinform.

[40] Ung P.M.-U., Rahman R., Schlessinger A. (2018). Redefining the protein kinase conformational space with machine learning. Cell Chem Biol.

[41] Sun T., Chen Y., Wen Y., Zhu Z., Li M. (2021). PremPLI: a machine learning model for predicting the effects of missense mutations on protein-ligand interactions. Commun Biol.

[42] Mou Z., Eakes J., Cooper C.J., Foster C.M., Standaert R.F., Podar M., Doktycz M.J., Parks J.M. (2021). Machine learning-based prediction of enzyme substrate scope: application to bacterial nitrilases. Proteins Struct Funct Bioinform.

[43] Robinson S.L., Smith M.D., Richman J.E., Aukema K.G., Wackett L.P. (2020). Machine learning-based prediction of activity and substrate specificity for OleA enzymes in the Thiolase superfamily. Synth Biol.

[44] Durairaj J., Melillo E., Bouwmeester H.J., Beekwilder J., de Ridder D., van Dijk A.D.J. (2021). Integrating structure-based machine learning and co-evolution to investigate specificity in plant sesquiterpene synthases. PLoS Comput Biol.

[45] He X.-h., You C.-z., Jiang H.-l., Jiang Y., Xu H.E., Cheng X. (2022). Alphafold2 versus experimental structures: evaluation on g protein-coupled receptors. Acta Pharmacol Sin.

[46] Timonina D., Sharapova Y., Švedas V., Suplatov D. (2021). Bioinformatic analysis of subfamily-specific regions in 3D-structures of homologs to study functional diversity and conformational plasticity in protein superfamilies. Comput Struct Biotechnol J.

[47] de Lima E.B., Júnior W.M., de Melo-Minardi R.C. (2016). Isofunctional protein subfamily detection using data integration and spectral clustering. PLoS Comput Biol.

[48] N. Ahalawat, J. Mondal, Resolving protein conformational plasticity and substrate binding through the lens of machine-learning, bioRxiv Preprint (Jan. 2022). 10.1101/2022.01.07.475334. 37068044

[49] A. Joshi, N. Haspel, E. González, Characterizing protein conformational spaces using dimensionality reduction and algebraic topology, bioRxiv Preprint (Nov. 2021). 10.1101/2021.11.16.468545.

[50] Peterson L.E. (2009). K-Nearest neighbor. Scholarpedia.

[51] Noble W.S. (2006). What is a support vector machine?. Nat Biotechnol.

[52] Rasmussen C.E., Bousquet O., von Luxburg U., Rätsch G. (2004). Advanced lectures on machine learning: ML summer schools 2003, Canberra, Australia, February 2 - 14, 2003, Tübingen, Germany, August 4 - 16, 2003, Revised Lectures, Lecture Notes in Computer Science.

[53] Breiman L. (2001). Random forests. Mach Learn.

[54] Friedman J.H. (2001). Greedy function approximation: a gradient boosting machine. Ann Stat.

[55] Cheng J., Randall A.Z., Sweredoski M.J., Baldi P. (2005). SCRATCH: a protein structure and structural feature prediction server. Nucleic Acids Res.

[56] Shen Y., Bax A. (2013). Protein backbone and sidechain torsion angles predicted from NMR chemical shifts using artificial neural networks. J Biomol NMR.

[57] Mataeimoghadam F., Newton M.A.H., Dehzangi A., Karim A., Jayaram B., Ranganathan S., Sattar A. (2020). Enhancing protein backbone angle prediction by using simpler models of deep neural networks. Sci Rep.

[58] Xu J. (2019). Distance-based protein folding powered by deep learning. Proc Natl Acad Sci USA.

[59] Jones D.T., Kandathil S.M. (2018). High precision in protein contact prediction using fully convolutional neural networks and minimal sequence features. Bioinformatics.

[60] Wang S., Sun S., Li Z., Zhang R., Xu J. (2017). Accurate de novo prediction of protein contact map by ultra-deep learning model. PLoS Comput Biol.

[61] Liu Y., Palmedo P., Ye Q., Berger B., Peng J. (2018). Enhancing evolutionary couplings with deep convolutional neural networks. Cell Syst.

[62] Ovchinnikov S., Park H., Varghese N., Huang P.-S., Pavlopoulos G.A., Kim D.E., Kamisetty H., Kyrpides N.C., Baker D. (2017). Protein structure determination using metagenome sequence data. Science.

[63] Kryshtafovych A., Schwede T., Topf M., Fidelis K., Moult J. (2019). Critical assessment of methods of protein structure prediction (CASP)—round XIII. Proteins Struct Funct Bioinform.

[64] Kryshtafovych A., Schwede T., Topf M., Fidelis K., Moult J. (2021). Critical assessment of methods of protein structure prediction (CASP)–round XIV. Proteins Struct Funct Bioinform.

[65] Kuhlman B., Bradley P. (2019). Advances in protein structure prediction and design. Nat Rev Mol Cell Biol.

[66] AlQuraishi M. (2021). Machine learning in protein structure prediction. Curr Opin Chem Biol.

[67] Gligorijević V., Renfrew P.D., Kosciolek T., Leman J.K., Berenberg D., Vatanen T., Chandler C., Taylor B.C., Fisk I.M., Vlamakis H., Xavier R.J., Knight R., Cho K., Bonneau R. (2021). Structure-based protein function prediction using graph convolutional networks. Nat Commun.

[68] Rauer C., Sen N., Waman V.P., Abbasian M., Orengo C.A. (2021). Computational approaches to predict protein functional families and functional sites. Curr Opin Struct Biol.

[69] Dana J.M., Gutmanas A., Tyagi N., Qi G., O’Donovan C., Martin M., Velankar S. (2019). SIFTS: updated Structure Integration with Function, Taxonomy and Sequences resource allows 40-fold increase in coverage of structure-based annotations for proteins. Nucleic Acids Res.

[70] Parthiban V., Gromiha M.M., Schomburg D. (2006). CUPSAT: prediction of protein stability upon point mutations. Nucleic Acids Res.

[71] Li B., Yang Y.T., Capra J.A., Gerstein M.B. (2020). Predicting changes in protein thermodynamic stability upon point mutation with deep 3D convolutional neural networks. PLoS Comput Biol.

[72] Masso M., Vaisman I.I. (2008). Accurate prediction of stability changes in protein mutants by combining machine learning with structure based computational mutagenesis. Bioinformatics.

[73] Quan L., Lv Q., Zhang Y. (2016). STRUM: Structure-based prediction of protein stability changes upon single-point mutation. Bioinformatics.

[74] Nikam R., Kulandaisamy A., Harini K., Sharma D., Gromiha M.M. (2021). ProThermDB: thermodynamic database for proteins and mutants revisited after 15 years. Nucleic Acids Res.

[75] R.J. Townshend, M. Vögele, P. Suriana, A. Derry, A. Powers, Y. Laloudakis, S. Balachandar, B. Jing, B. Anderson, S. Eismann, et al., Atom3d: Tasks on molecules in three dimensions, arXiv preprint arXiv:2012.04035 (2020).

[76] Naderi M., Lemoine J.M., Govindaraj R.G., Kana O.Z., Feinstein W.P., Brylinski M. (2019). Binding site matching in rational drug design: algorithms and applications. Brief Bioinform.

[77] Pu L., Govindaraj R.G., Lemoine J.M., Wu H.-C., Brylinski M. (2019). DeepDrug3D: classification of ligand-binding pockets in proteins with a convolutional neural network. PLoS Comput Biol.

[78] Brylinski M. (2014). eMatchSite: Sequence order-independent structure alignments of ligand binding pockets in protein models. PLoS Comput Biol.

[79] Ragoza M., Hochuli J., Idrobo E., Sunseri J., Koes D.R. (2017). Protein-ligand scoring with convolutional neural networks. J Chem Inf Model.

[80] Pagès G., Charmettant B., Grudinin S. (2019). Protein model quality assessment using 3D oriented convolutional neural networks. Bioinformatics.

[81] Shen C., Ding J., Wang Z., Cao D., Ding X., Hou T. (2020). From machine learning to deep learning: advances in scoring functions for protein-ligand docking. WIREs Comput Mol Sci.

[82] Hiranuma N., Park H., Baek M., Anishchenko I., Dauparas J., Baker D. (2021). Improved protein structure refinement guided by deep learning based accuracy estimation. Nat Commun.

[83] Kryshtafovych A., Monastyrskyy B., Fidelis K. (2016). CASP11 statistics and the prediction center evaluation system. Proteins Struct Funct Bioinform.

[84] Townshend R., Bedi R., Suriana P., Dror R. (2019). End-to-end Learning on 3D protein structure for interface prediction. Adv Neural Inf Process Syst.

[85] Sanchez-Garcia R., Sorzano C.O.S., Carazo J.M. (2019). A method for the prediction of partner-specific protein-protein interfaces. Bioinformatics.

[86] Gainza P., Sverrisson F., Monti F., Rodolà E., Boscaini D., Bronstein M.M., Correia B.E. (2020). Deciphering interaction fingerprints from protein molecular surfaces using geometric deep learning. Nat Methods.

[87] U. Ghani, I. Desta, A. Jindal, O. Khan, G. Jones, S. Kotelnikov, D. Padhorny, S. Vajda, D. Kozakov, Improved docking of protein models by a combination of alphafold2 and cluspro, bioRxiv Preprint (Sep. 2021). 10.1101/2021.09.07.459290.

[88] Bendell C.J., Liu S., Aumentado-Armstrong T., Istrate B., Cernek P.T., Khan S., Picioreanu S., Zhao M., Murgita R.A. (2014). Transient protein-protein interface prediction: datasets, features, algorithms, and the rad-t predictor. BMC Bioinform.

[89] Das S., Chakrabarti S. (2021). Classification and prediction of protein-protein interaction interface using machine learning algorithm. Sci Rep.

[90] Xu Q., Dunbrack R.L. (2020). Protcid: a data resource for structural information on protein interactions. Nat Commun.

[91] Vreven T., Moal I.H., Vangone A., Pierce B.G., Kastritis P.L., Torchala M., Chaleil R., Jiménez-García B., Bates P.A., Fernandez-Recio J., Bonvin A.M.J.J., Weng Z. (2015). Updates to the integrated protein-protein interaction benchmarks: Docking Benchmark Version 5 and Affinity Benchmark Version 2. J Mol Biol.

[92] Kundrotas P.J., Anishchenko I., Dauzhenka T., Kotthoff I., Mnevets D., Copeland M.M., Vakser I.A. (2018). Dockground: a comprehensive data resource for modeling of protein complexes. Protein Sci.

[93] A. Morehead, C. Chen, A. Sedova, Dips-plus: The enhanced database of interacting protein structures for interface prediction, arXiv preprint arXiv:2106.04362 (2021). 10.1038/s41597-023-02409-3 PMC10400622 37537186

[94] Jiménez J., Doerr S., Martínez-Rosell G., Rose A.S., Fabritiis G.De (2017). DeepSite: Protein-binding site predictor using 3D-convolutional neural networks. Bioinformatics.

[95] Kozlovskii I., Popov P. (2020). Spatiotemporal identification of druggable binding sites using deep learning. Commun Biol.

[96] Krivák R., Hoksza D. (2018). P2Rank: machine learning based tool for rapid and accurate prediction of ligand binding sites from protein structure. J Cheminfor.

[97] Desaphy J., Bret G., Rognan D., Kellenberger E. (2015). sc-PDB: a 3D-database of ligandable binding sites—10 years on. Nucleic Acids Res.

[98] Roy A., Yang J., Zhang Y. (2012). COFACTOR: an accurate comparative algorithm for structure-based protein function annotation. Nucleic Acids Res.

[99] Schmidtke P., Souaille C., Estienne F., Baurin N., Kroemer R.T. (2010). Large-scale comparison of four binding site detection algorithms. J Chem Inf Model.

[100] Mészáros B., Erdős G., Dosztányi Z. (2018). IUPred2A: Context-dependent prediction of protein disorder as a function of redox State and protein binding. Nucleic Acids Res.

[101] McGuffin L.J. (2008). Intrinsic disorder prediction from the analysis of multiple protein fold recognition models. Bioinformatics.

[102] Schad E., Fichó E., Pancsa R., Simon I., Dosztányi Z., Mészáros B. (2018). DIBS: a repository of disordered binding sites mediating interactions with ordered proteins. Bioinformatics.

[103] Piovesan D., Tabaro F., Mičetić I., Necci M., Quaglia F., Oldfield C.J., Aspromonte M.C., Davey N.E., Davidović R., Dosztányi Z., Elofsson A., Gasparini A., Hatos A., Kajava A.V., Kalmar L., Leonardi E., Lazar T., Macedo-Ribeiro S., Macossay-Castillo M., Meszaros A., Minervini G., Murvai N., Pujols J., Roche D.B., Salladini E., Schad E., Schramm A., Szabo B., Tantos A., Tonello F., Tsirigos K.D., Veljković N., Ventura S., Vranken W., Warholm P., Uversky V.N., Dunker A., Longhi S., Tompa P., Tosatto S.C. (2017). DisProt 7.0: a major update of the database of disordered proteins. Nucleic Acids Res.

[104] Wass M.N., Fuentes G., Pons C., Pazos F., Valencia A. (2011). Towards the prediction of protein interaction partners using physical docking. Mol Syst Biol.

[105] Zhang Q.C., Petrey D., Deng L., Qiang L., Shi Y., Thu C.A., Bisikirska B., Lefebvre C., Accili D., Hunter T., Maniatis T., Califano A., Honig B. (2012). Structure-based prediction of protein-protein interactions on a genome-wide scale. Nature.

[106] I.R. Humphreys, J. Pei, M. Baek, A. Krishnakumar, I. Anishchenko, S. Ovchinnikov, J. Zhang, T.J. Ness, S. Banjade, S. Bagde, V.G. Stancheva, X.-H. Li, K. Liu, Z. Zheng, D.J. Barrero, U. Roy, I.S. Fernández, B. Szakal, D. Branzei, E.C. Greene, S. Biggins, S. Keeney, E.A. Miller, J.C. Fromme, T.L. Hendrickson, Q. Cong, D. Baker, Structures of core eukaryotic protein complexes, bioRxiv Preprint (Sep. 2021). 10.1101/2021.09.30.462231. PMC7612107 34762488

[107] Salwinski L., Miller C.S., Smith A.J., Pettit F.K., Bowie J.U., Eisenberg D. (2004). The database of interacting proteins: 2004 update. Nucleic Acids Res.

[108] Szklarczyk D., Gable A.L., Nastou K.C., Lyon D., Kirsch R., Pyysalo S., Doncheva N.T., Legeay M., Fang T., Bork P., Jensen L.J., von Mering C. (2021). The STRING database in 2021: customizable protein-protein networks, and functional characterization of user-uploaded gene/measurement sets. Nucleic Acids Res.

[109] Peri S., Navarro J.D., Amanchy R., Kristiansen T.Z., Jonnalagadda C.K., Surendranath V., Niranjan V., Muthusamy B., Gandhi T.K.B., Gronborg M., Ibarrola N., Deshpande N., Shanker K., Shivashankar H.N., Rashmi B.P., Ramya M.A., Zhao Z., Chandrika K.N., Padma N., Harsha H.C., Yatish A.J., Kavitha M.P., Menezes M., Choudhury D.R., Suresh S., Ghosh N., Saravana R., Chandran S., Krishna S., Joy M., Anand S.K., Madavan V., Joseph A., Wong G.W., Schiemann W.P., Constantinescu S.N., Huang L., Khosravi-Far R., Steen H., Tewari M., Ghaffari S., Blobe G.C., Dang C.V., Garcia J.G.N., Pevsner J., Jensen O.N., Roepstorff P., Deshpande K.S., Chinnaiyan A.M., Hamosh A., Chakravarti A., Pandey A. (2003). Development of human protein reference database as an initial platform for approaching systems biology in humans. Genome Res.

[110] Oughtred R., Rust J., Chang C., Breitkreutz B.-J., Stark C., Willems A., Boucher L., Leung G., Kolas N., Zhang F., Dolma S., Coulombe-Huntington J., Chatr-Aryamontri A., Dolinski K., Tyers M. (2021). The BioGRID database: a comprehensive biomedical resource of curated protein, genetic, and chemical interactions. Protein Sci.

[111] Kumar R., Nanduri B. (2010). HPIDB - a unified resource for host-pathogen interactions. BMC Bioinform.

[112] Zhang N., Chen Y., Lu H., Zhao F., Alvarez R.V., Goncearenco A., Panchenko A.R., Li M. (2020). MutaBind2: Predicting the impacts of single and multiple mutations on protein-protein interactions. iScience.

[113] Liu X., Luo Y., Li P., Song S., Peng J. (2021). Deep geometric representations for modeling effects of mutations on protein-protein binding affinity. PLoS Comput Biol.

[114] Geng C., Vangone A., Folkers G.E., Xue L.C., Bonvin A.M.J.J. (2019). iSEE: Interface structure, evolution, and energy-based machine learning predictor of binding affinity changes upon mutations. Proteins Struct Funct Bioinform.

[115] Jankauskaitė J., Jiménez-García B., Dapkunas J., Fernández-Recio J., Moal I.H. (2019). SKEMPI 2.0: an updated benchmark of changes in protein-protein binding energy, kinetics and thermodynamics upon mutation. Bioinformatics.

[116] Jiménez J., Škalič M., Martínez-Rosell G., Fabritiis G.De (2018). KDEEP: Protein-ligand absolute binding affinity prediction via 3D-convolutional neural networks. J Chem Inf Model.

[117] Ahmed A., Mam B., Sowdhamini R. (2021). DEELIG: A deep learning approach to predict protein-ligand binding affinity. Bioinform Biol Insights.

[118] Ballester P.J., Mitchell J.B.O. (2010). A machine learning approach to predicting protein-ligand binding affinity with applications to molecular docking. Bioinformatics.

[119] Boyles F., Deane C.M., Morris G.M. (2021). Learning from docked ligands: Ligand-based features rescue structure-based scoring functions when trained on docked poses. J Chem Inf Model.

[120] Kundu I., Paul G., Banerjee R. (2018). A machine learning approach towards the prediction of protein- ligand binding affinity based on fundamental molecular properties. RSC Adv.

[121] Li H., Leung K.-S., Wong M.-H., Ballester P.J. (2015). Improving AutoDock Vina using Random Forest: the growing accuracy of binding affinity prediction by the effective exploitation of larger data sets. Mol Inf.

[122] S. Li, J. Zhou, T. Xu, L. Huang, F. Wang, H. Xiong, W. Huang, D. Dou, H. Xiong, Structure-aware interactive graph neural networks for the prediction of protein-ligand binding affinity, in: Proceedings of the 27th ACM SIGKDD Conference on Knowledge Discovery & Data Mining, ACM, Virtual Event Singapore, 2021, pp.975–985. 10.1145/3447548.3467311.

[123] Stepniewska-Dziubinska M.M., Zielenkiewicz P., Siedlecki P. (2018). Development and evaluation of a deep learning model for protein- ligand binding affinity prediction. Bioinformatics.

[124] Wójcikowski M., Kukiełka M., Stepniewska-Dziubinska M.M., Siedlecki P. (2019). Development of a Protein-Ligand Extended Connectivity (PLEC) fingerprint and its application for binding affinity predictions. Bioinformatics.

[125] Liu Z., Li Y., Han L., Li J., Liu J., Zhao Z., Nie W., Liu Y., Wang R. (2015). PDB-wide collection of binding data: current status of the PDBbind database. Bioinformatics.

[126] Hu L., Benson M.L., Smith R.D., Lerner M.G., Carlson H.A. (2005). Binding MOAD (Mother Of All Databases). Proteins Struct Funct Bioinform.

[127] Mysinger M.M., Carchia M., Irwin J.J., Shoichet B.K. (2012). Directory of useful decoys, enhanced (dud-e): better ligands and decoys for better benchmarking. J Med Chem.

[128] R. Evans, M. O’Neill, A. Pritzel, N. Antropova, A. Senior, T. Green, A. Žídek, R. Bates, S. Blackwell, J. Yim, O. Ronneberger, S. Bodenstein, M. Zielinski, A. Bridgland, A. Potapenko, A. Cowie, K. Tunyasuvunakool, R. Jain, E. Clancy, P. Kohli, J. Jumper, D. Hassabis, Protein complex prediction with AlphaFold-Multimer, bioRxiv Preprint (Oct. 2021). 10.1101/2021.10.04.463034.

[129] Bryant P., Pozzati G., Elofsson A. (2022). Improved prediction of protein-protein interactions using AlphaFold2. Nat Commun.

[130] P. Bryant, G. Pozzati, W. Zhu, A. Shenoy, P. Kundrotas, A. Elofsson, Predicting the structure of large protein complexes using AlphaFold and Monte Carlo tree search, Nat Commun 13(1) (2022) 6028. 10.1038/s41467-022-33729-4. PMC9556563 36224222

[131] Yin R., Feng B.Y., Varshney A., Pierce B.G. (2022). Benchmarking AlphaFold for protein complex modeling reveals accuracy determinants. Protein Sci.

[132] M. Baek, R. McHugh, I. Anishchenko, D. Baker, F. DiMaio, Accurate prediction of nucleic acid and protein-nucleic acid complexes using rosettafoldna, bioRxiv (2022). 10.1101/2022.09.09.507333. PMC10776382 37996753

[133] Lima A.N., Philot E.A., Trossini G.H.G., Scott L.P.B., Maltarollo V.G., Honorio K.M. (2016). Use of machine learning approaches for novel drug discovery. Expert Opin Drug Discov.

[134] Zhao J., Cao Y., Zhang L. (2020). Exploring the computational methods for protein-ligand binding site prediction. Comput Struct Biotechnol J.

[135] Lee M., Kim D. (2012). Large-scale reverse docking profiles and their applications. BMC Bioinform.

[136] Grinter S.Z., Liang Y., Huang S.-Y., Hyder S.M., Zou X. (2011). An inverse docking approach for identifying new potential anti-cancer targets. J Mol Graph Model.

[137] Fernández A. (2020). Artificial intelligence teaches drugs to target proteins by tackling the induced folding problem. Mol Pharm.

[138] Z. Xu, O.R. Wauchope, A.T. Frank, Navigating chemical space by interfacing generative artificial intelligence and molecular docking, J Chem Inf Model 61(11) (2021) 5589–5600. 10.1021/acs.jcim.1c00746. 34633194

[139] P. Drotár, A.R. Jamasb, B. Day, C. Cangea, P. Liò, Structure-aware generation of drug-like molecules, arXiv Preprint (Nov. 2021.

[140] Wong F., Krishnan A., Zheng E.J., Stärk H., Manson A.L., Earl A.M., Jaakkola T., Collins J.J. (2022). Benchmarking alphafold-enabled molecular docking predictions for antibiotic discovery. Mol Syst Biol.

[141] N. Sen, I. Anishchenko, N. Bordin, I. Sillitoe, S. Velankar, D. Baker, C. Orengo, Characterizing disease-associated human proteins without available protein structures or homologues, bioRxiv Preprint (Nov. 2021). 10.1101/2021.11.17.468998.

[142] Pak M.A., Ivankov D.N. (2022). Best templates outperform homology models in predicting the impact of mutations on protein stability. Bioinform Btac.

[143] M.A. Pak, K.A. Markhieva, M.S. Novikova, D.S. Petrov, I.S. Vorobyev, E.S. Maksimova, F.A. Kondrashov, D.N. Ivankov, Using alphafold to predict the impact of single mutations on protein stability and function, BioRxiv (2021). 10.1371/journal.pone.0282689 PMC10019719 36928239

[144] C. Norn, B.I.M. Wicky, D. Juergens, S. Liu, D. Kim, B. Koepnick, I. Anishchenko, F. Players, D. Baker, S. Ovchinnikov, Protein sequence design by explicit energy landscape optimization, bioRxiv (2020). 10.1101/2020.07.23.218917.

[145] D. Tischer, S. Lisanza, J. Wang, R. Dong, I. Anishchenko, L.F. Milles, S. Ovchinnikov, D. Baker, Design of proteins presenting discontinuous functional sites using deep learning, bioRxiv (2020). 10.1101/2020.11.29.402743.

[146] J. Wang, S. Lisanza, D. Juergens, D. Tischer, I. Anishchenko, M. Baek, J.L. Watson, J.H. Chun, L.F. Milles, J. Dauparas, M. Expòsit, W. Yang, A. Saragovi, S. Ovchinnikov, D. Baker, Deep learning methods for designing proteins scaffolding functional sites, bioRxiv Preprint (Nov. 2021). 10.1101/2021.11.10.468128.

[147] Anishchenko I., Pellock S.J., Chidyausiku T.M., Ramelot T.A., Ovchinnikov S., Hao J., Bafna K., Norn C., Kang A., Bera A.K., DiMaio F., Carter L., Chow C.M., Montelione G.T., Baker D. (2021). De novo protein design by deep network hallucination. Nature.

[148] Lin Y.-R., Koga N., Tatsumi-Koga R., Liu G., Clouser A.F., Montelione G.T., Baker D. (2015). Control over overall shape and size in de novo designed proteins. Proc Natl Acad Sci USA.

[149] Marcos E., Chidyausiku T.M., McShan A.C., Evangelidis T., Nerli S., Carter L., Nivón L.G., Davis A., Oberdorfer G., Tripsianes K., Sgourakis N.G., Baker D. (2018). De novo design of a non-local *β*-sheet protein with high stability and accuracy. Nat Struct Mol Biol.

[150] Baker D. (2019). What has de novo protein design taught us about protein folding and biophysics?. Protein Sci.

[151] N. Ferruz, M. Heinzinger, M. Akdel, A. Goncearenco, L. Naef, C. Dallago, From sequence to function through structure: deep learning for protein design, bioRxiv (2022). 10.1016/j.csbj.2022.11.014 PMC9755234 36544476

[152] Ward J.J., Sodhi J.S., McGuffin L.J., Buxton B.F., Jones D.T. (2004). Prediction and functional analysis of native disorder in proteins from the three kingdoms of life. J Mol Biol.

[153] A. Gupta, S. Dey, H.-X. Zhou, Artificial Intelligence Guided Conformational Mining of Intrinsically Disordered Proteins, bioRxiv Preprint(Nov. 2021). 10.1101/2021.11.21.469457. PMC9209487 35725761

[154] Budowski-Tal I., Nov Y., Kolodny R. (2010). FragBag, an accurate representation of protein structure, retrieves structural neighbors from the entire PDB quickly and accurately. Proc Natl Acad Sci USA.

[155] Liu Y., Ye Q., Wang L., Peng J. (2018). Learning structural motif representations for efficient protein structure search. Bioinformatics.

[156] Guzenko D., Burley S.K., Duarte J.M. (2020). Real time structural search of the protein data bank. PLoS Comput Biol.

[157] T. Aderinwale, V. Bharadwaj, C. Christoffer, G. Terashi, Z. Zhang, R. Jahandideh, Y. Kagaya, D. Kihara, Real-Time Structure Search and Structure Classification for AlphaFold Protein Models, bioRxiv Preprint (Oct. 2021). 10.1101/2021.10.21.465371. PMC8983703 35383281

[bib158] 158Foldseek: fast and accurate protein structure search bioRxiv 10.1101/2022.02.07.479398v4

[159] N. Bordin, I. Sillitoe, V. Nallapareddy, C. Rauer, S.D. Lam, V.P. Waman, N. Sen, M. Heinzinger, M. Littmann, S. Kim, S. Velankar, M. Steinegger, B. Rost, C. Orengo, AlphaFold2 reveals commonalities and novelties in protein structure space for 21 model organisms, pages: 2022.06.02.494367 Section: New Results (Jun. 2022). 10.1101/2022.06.02.494367. PMC9908985 36755055

[160] Niu B., Scott A.D., Sengupta S., Bailey M.H., Batra P., Ning J., Wyczalkowski M.A., Liang W.-W., Zhang Q., McLellan M.D., Sun S.Q., Tripathi P., Lou C., Ye K., Mashl R.J., Wallis J., Wendl M.C., Chen F., Ding L. (2016). Protein-structure-guided discovery of functional mutations across 19 cancer types. Nat Genet.

[161] Berliner N., Teyra J., Çolak R., Lopez S.G., Kim P.M. (2014). Combining structural modeling with ensemble machine learning to accurately predict protein fold stability and binding affinity effects upon mutation. PLoS One.

[162] Terwilliger T.C., Liebschner D., Croll T.I., Williams C.J., McCoy A.J., Poon B.K., Afonine P.V., Oeffner R.D., Richardson J.S., Read R.J., Adams P.D. (2022). AlphaFold predictions: great hypotheses but no match for experiment, preprint. Biochemistry.

[163] Hubbard S.J., Thornton J.M. (1993). naccess, computer program, department of biochemistry and molecular biology. Univ Coll Lond.

[164] Mihel J., Šikić M., Tomić S., Jeren B., Vlahoviček K. (2008). Psaia-protein structure and interaction analyzer. BMC Struct Biol.

[165] Mitternacht S. (2016). Freesasa: An open source c library for solvent accessible surface area calculations. F1000Research.

[166] Touw W.G., Baakman C., Black J., Te Beek T.A., Krieger E., Joosten R.P., Vriend G. (2015). A series of pdb-related databanks for everyday needs. Nucleic Acids Res.

[167] Ruiz-Blanco Y.B., Paz W., Green J., Marrero-Ponce Y. (2015). Protdcal: A program to compute general-purpose-numerical descriptors for sequences and 3d-structures of proteins. BMC Bioinform.

[168] Cock P.J., Antao T., Chang J.T., Chapman B.A., Cox C.J., Dalke A., Friedberg I., Hamelryck T., Kauff F., Wilczynski B. (2009). Biopython: freely available python tools for computational molecular biology and bioinformatics. Bioinformatics.

[169] Sanner M.F., Olson A.J., Spehner J.-C. (1996). Reduced surface: an efficient way to compute molecular surfaces. Biopolymers.

[170] R.J. Gowers, M. Linke, J. Barnoud, T.J.E. Reddy, M.N. Melo, S.L. Seyler, J. Domanski, D.L. Dotson, S. Buchoux, I.M. Kenney, et al., Mdanalysis: a python package for the rapid analysis of molecular dynamics simulations, Tech. rep., Los Alamos National Lab. (LANL), Los Alamos, NM (United States) (2019).

[171] Buß O., Rudat J., Ochsenreither K. (2018). Foldx as protein engineering tool: better than random based approaches?. Comput Struct Biotechnol J.

[172] Alford R.F., Leaver-Fay A., Jeliazkov J.R., O’Meara M.J., DiMaio F.P., Park H., Shapovalov M.V., Renfrew P.D., Mulligan V.K., Kappel K. (2017). The rosetta all-atom energy function for macromolecular modeling and design. J Chem Theory Comput.

[173] Baker N.A., Sept D., Joseph S., Holst M.J., McCammon J.A. (2001). Electrostatics of nanosystems: application to microtubules and the ribosome. Proc Natl Acad Sci USA.

[174] Ward J.J., McGuffin L.J., Bryson K., Buxton B.F., Jones D.T. (2004). The disopred server for the prediction of protein disorder. Bioinformatics.

[175] Bakan A., Meireles L.M., Bahar I. (2011). Prody: protein dynamics inferred from theory and experiments. Bioinformatics.

[176] Mikulska-Ruminska K., Kulik A.J., Kaya C., BenAdiba C., Dietler G., Nowak W., Bahar I. (2017). Mechstiff: A new tool for evaluating stress-induced dynamics and application to cell adhesion proteins. Biophys J.

[177] Atilgan C., Atilgan A.R. (2009). Perturbation-response scanning reveals ligand entry-exit mechanisms of ferric binding protein. PLoS Comput Biol.

[178] Shegay M.V., Suplatov D.A., Popova N.N., Švedas V.K., Voevodin V.V. (2019). parMATT: Parallel multiple alignment of protein 3D-structures with translations and twists for distributed-memory systems. Bioinformatics.

[179] J. Durairaj, M. Akdel, D. de Ridder, A.D. van Dijk, Fast and adaptive protein structure representations for machine learning, bioRxiv Preprint (Apr. 2021). 10.1101/2021.04.07.438777.

[180] Shegay M.V., Švedas V.K., Voevodin V.V., Suplatov D.A., Popova N.N. (2021). Guide tree optimization with genetic algorithm to improve multiple protein 3D-structure alignment. Bioinformatics.

[181] Ezkurdia I., Bartoli L., Fariselli P., Casadio R., Valencia A., Tress M.L. (2009). Progress and challenges in predicting protein- protein interaction sites. Brief Bioinform.

[182] Poupon A. (2004). Voronoi and voronoi-related tessellations in studies of protein structure and interaction. Curr Opin Struct Biol.

[183] Pan Y., Wang Z., Zhan W., Deng L. (2018). Computational identification of binding energy hot spots in protein-RNA complexes using an ensemble approach. Bioinformatics.

[184] Igashov I., Olechnovič K., Kadukova M., Venclovas Č., Grudinin S. (2021). VoroCNN: Deep convolutional neural network built on 3D voronoi tessellation of protein structures. Bioinformatics.

[185] Bernauer J., Bahadur R.P., Rodier F., Janin J., Poupon A. (2008). DiMoVo: A voronoi tessellation-based method for discriminating crystallographic and biological protein– protein interactions. Bioinformatics.

[186] Durairaj J., Akdel M., de Ridder D., van Dijk A.D.J. (2020). Geometricus represents protein structures as shape-mers derived from moment invariants. Bioinformatics.

[187] Kihara D., Sael L., Chikhi R., Esquivel-Rodriguez J. (2011). Molecular surface representation Using 3D Zernike descriptors for protein shape comparison and docking. Curr Protein Peptide Sci.

[188] Yin S., Proctor E.A., Lugovskoy A.A., Dokholyan N.V. (2009). Fast screening of protein surfaces using geometric invariant fingerprints. Proc Natl Acad Sci USA.

[189] Namrata A., Po-Ssu H. (2018). Generative modeling for protein structures. Adv Neural Inf Process Syst.

[190] Jiang M., Li Z., Zhang S., Wang S., Wang X., Yuan Q., Wei Z. (2020). Drug- target affinity prediction using graph neural network and contact maps. RSC Adv.

[191] Wang X., Flannery S.T., Kihara D. (2021). Protein docking model evaluation by graph neural networks. Front Mol Biosci.

[192] Strokach A., Becerra D., Corbi-Verge C., Perez-Riba A., Kim P.M. (2020). Fast and flexible protein design using deep graph neural networks. Cell Syst.

[193] Ingraham J., Garg V., Barzilay R., Jaakkola T. (2019). Generative models for graph-based protein design. Adv Neural Inf Process Syst.

[194] Q. Yuan, S. Chen, J. Rao, S. Zheng, H. Zhao, Y. Yang, AlphaFold2-aware protein-DNA binding site prediction using graph transformer, bioRxiv Preprint (Dec. 2021). 10.1101/2021.08.25.457661. 35039821

[195] A.R. Jamasb, R. Viñas, E.J. Ma, C. Harris, K. Huang, D. Hall, P. Lió, T.L. Blundell, Graphein - a Python library for geometric deep learning and network analysis on protein structures and interaction networks, bioRxiv Preprint (Oct. 2021). 10.1101/2020.07.15.204701.

[196] Somnath V.R., Bunne C., Krause A. (2021). Multi-scale representation learning on proteins. Adv Neural Inf Process Syst.

[197] Lim J., Ryu S., Park K., Choe Y.J., Ham J., Kim W.Y. (2019). Predicting drug-target interaction using a novel graph neural network with 3D structure-embedded graph representation. J Chem Inf Model.

[198] Morrone J.A., Weber J.K., Huynh T., Luo H., Cornell W.D. (2020). Combining docking pose rank and structure with deep learning improves protein-ligand binding mode prediction over a baseline docking approach. J Chem Inf Model.

[199] Sunseri J., King J.E., Francoeur P.G., Koes D.R. (2019). Convolutional neural network scoring and minimization in the D3R 2017 community challenge. J Comput Aided Mol Des.

[200] Wu Z., Ramsundar B., Feinberg E.N., Gomes J., Geniesse C., Pappu A.S., Leswing K., Pande V. (2018). MoleculeNet: A benchmark for molecular machine learning. Chem Sci.

[201] Qin T., Zhu Z., Wang X.S., Xia J., Wu S. (2021). Computational representations of protein- ligand interfaces for structure-based virtual screening. Expert Opin Drug Discov.

[202] Alley E.C., Khimulya G., Biswas S., AlQuraishi M., Church G.M. (2019). Unified rational protein engineering with sequence-based deep representation learning. Nat Methods.

[203] T. Bepler, B. Berger, Learning protein sequence embeddings using information from structure, arXiv Preprint (Oct. 2019). arXiv:1902.08661. PMC12947628 41768824

[204] Heinzinger M., Elnaggar A., Wang Y., Dallago C., Nechaev D., Matthes F., Rost B. (2019). Modeling aspects of the language of life through transfer-learning protein sequences. BMC Bioinform.

[205] Rives A., Meier J., Sercu T., Goyal S., Lin Z., Liu J., Guo D., Ott M., Zitnick C.L., Ma J., Fergus R. (2021). Biological structure and function emerge from scaling unsupervised learning to 250 million protein sequences. Proc Natl Acad Sci USA.

[206] Mansoor S., Baek M., Madan U., Horvitz E. (2021). Toward more general embeddings for protein design: harnessing joint representations of sequence and structure. bioRxiv Preprint.

[207] P. Hermosilla, T. Ropinski, Contrastive representation learning for 3d protein structures, arXiv preprint arXiv:2205.15675 (2022).

[208] C. Chen, Y. Zha, D. Zhu, K. Ning, X. Cui, Hydrogen bonds meet self-attention: all you need for general-purpose protein structure embedding, bioRxiv Preprint (Aug. 2021). 10.1101/2021.01.31.428935.

[209] A. Vaswani, N. Shazeer, N. Parmar, J. Uszkoreit, L. Jones, A.N. Gomez, Ł. Kaiser, I. Polosukhin, Attention is all you need, in: Advances in neural information processing systems, 2017, pp.5998–6008.

[210] F. Sverrisson, J. Feydy, B.E. Correia, M.M. Bronstein, Fast end-to-end learning on protein surfaces, bioRxiv Preprint (Dec. 2020). 10.1101/2020.12.28.424589.

[211] G. Corso, H. Stärk, B. Jing, R. Barzilay, T. Jaakkola, DiffDock:Diffusion Steps, Twists, and Turns for Molecular Docking, arXiv:2210.01776 [physics, q-bio](Oct. 2022). 10.48550/arXiv.2210.01776.

[212] O.-E. Ganea, X. Huang, C. Bunne, Y. Bian, R. Barzilay, T. Jaakkola, A. Krause, Independent SE(3)-equivariant models for end-to-end rigid protein docking, arXiv:2111.07786 [cs] (Mar. 2022). 10.48550/arXiv.2111.07786.

[213] A. Schneuing, Y. Du, C. Harris, A. Jamasb, I. Igashov, W. Du, T. Blundell, P. Lió, C. Gomes, M. Welling, M. Bronstein, B. Correia, Structure-based drug design with equivariant diffusion models, arXiv:2210.13695 [cs, q-bio](Oct. 2022). 10.48550/arXiv.2210.13695. PMC11659159 39653846

[214] Kim P.T., Winter R., Clevert D.-A. (2021). Unsupervised representation learning for proteochemometric modeling. Int J Mol Sci.

[215] Villegas-Morcillo A., Makrodimitris S., van Ham R.C.H.J., Gomez A.M., Sanchez V., Reinders M.J.T. (2021). Unsupervised protein embeddings outperform hand-crafted sequence and structure features at predicting molecular function. Bioinformatics.

[216] S. Sledzieski, R. Singh, L. Cowen, B. Berger, Sequence-based prediction of protein-protein interactions: a structure-aware interpretable deep learning model, bioRxiv (2021). 10.1101/2021.01.22.427866. PMC8586911 34536380

[217] M. Heinzinger, M. Littmann, I. Sillitoe, N. Bordin, C. Orengo, B. Rost, Contrastive learning on protein embeddings enlightens midnight zone at lightning speed, bioRxiv Preprint (Nov. 2021). 10.1101/2021.11.14.468528. PMC9188115 35702380

[218] Y. Zhang, P. Li, F. Pan, H. Liu, P. Hong, X. Liu, J. Zhang, Applications of AlphaFold beyond protein structure prediction, bioRxiv Preprint (Dec. 2021). 10.1101/2021.11.03.467194.

[219] Waterhouse A., Bertoni M., Bienert S., Studer G., Tauriello G., Gumienny R., Heer F.T., de Beer T.A.P., Rempfer C., Bordoli L., Lepore R., Schwede T. (2018). SWISS-MODEL: homology modelling of protein structures and complexes. Nucleic Acids Res.

[220] M. Mirdita, S. Ovchinnikov, M. Steinegger, ColabFold - Making protein folding accessible to all, bioRxiv Preprint (Aug. 2021). 10.1101/2021.08.15.456425. PMC9184281 35637307

[221] Weißenow K., Heinzinger M., Rost B. (2022). Protein language-model embeddings for fast, accurate, and alignment-free protein structure prediction. Structure.

[222] AlQuraishi M., Sorger P.K. (2021). Differentiable biology: using deep learning for biophysics-based and data-driven modeling of molecular mechanisms. Nat Methods.

[223] Ferruz N., Heinzinger M., Akdel M., Goncearenco A., Naef L., Dallago C. (2023). From sequence to function through structure: deep learning for protein design. Comput Struct Biotechnol J.

[224] Dauparas J., Anishchenko I., Bennett N., Bai H., Ragotte R.J., Milles L.F., Wicky B.I.M., Courbet A., de Haas R.J., Bethel N., Leung P.J.Y., Huddy T.F., Pellock S., Tischer D., Chan F., Koepnick B., Nguyen H., Kang A., Sankaran B., Bera A.K., King N.P., Baker D. (2022). Robust deep learning-based protein sequence design using ProteinMPNN. Science.

[225] J.L. Watson, D. Juergens, N.R. Bennett, B.L. Trippe, J. Yim, H.E. Eisenach, W. Ahern, A.J. Borst, R.J. Ragotte, L.F. Milles, B.I.M. Wicky, N. Hanikel, S.J. Pellock, A. Courbet, W. Sheffler, J. Wang, P. Venkatesh, I. Sappington, S.V. Torres, A. Lauko, V.D. Bortoli, E. Mathieu, R. Barzilay, T.S. Jaakkola, F. DiMaio, M. Baek, D. Baker, Broadly applicable and accurate protein design by integrating structure prediction networks and diffusion generative models, pages: 2022.12.09.519842 Section: New Results (Dec. 2022). 10.1101/2022.12.09.519842.

[226] Kmiecik S., Kouza M., Badaczewska-Dawid A.E., Kloczkowski A., Kolinski A. (2018). Modeling of protein structural flexibility and large-scale dynamics: coarse-grained simulations and Elastic Network Models. Int J Mol Sci.

[227] Hollingsworth S.A., Dror R.O. (2018). Molecular dynamics simulation for all. Neuron.

[228] Quesne M.G., Borowski T., de Visser S.P. (2016). Quantum mechanics/molecular mechanics modeling of enzymatic processes: caveats and breakthroughs. Chem Eur J.

[229] Atilgan A.R., Durell S.R., Jernigan R.L., Demirel M.C., Keskin O., Bahar I. (2001). Anisotropy of fluctuation dynamics of proteins with an Elastic Network Model. Biophys J.

[230] Jamroz M., Orozco M., Kolinski A., Kmiecik S. (2013). Consistent view of protein fluctuations from all-atom molecular dynamics and coarse-grained dynamics with knowledge-based force-field. J Chem Theory Comput.

[231] Frappier V., Najmanovich R.J. (2014). A coarse-grained elastic network atom contact model and its use in the simulation of protein dynamics and the prediction of the effect of mutations. PLoS Comput Biol.

[232] Tekpinar M., Zheng W. (2010). Predicting order of conformational changes during protein conformational transitions using an interpolated Elastic Network Model. Proteins Struct Funct Genet.

[233] Kmiecik S., Gront D., Kouza M., Kolinski A. (2012). From coarse-grained to atomic-level characterization of protein dynamics: transition state for the folding of B domain of protein A. J Phys Chem B.

[234] Mahajan S., Sanejouand Y.-H. (2015). On the relationship between low-frequency normal modes and the large-scale conformational changes of proteins. Arch Biochem Biophys.

[235] Yang L., Song G., Jernigan R.L. (2007). How well can we understand large-scale protein motions using normal modes of Elastic Network Models?. Biophys J.

[236] Takada S., Kanada R., Tan C., Terakawa T., Li W., Kenzaki H. (2015). Modeling structural dynamics of biomolecular complexes by coarse-grained molecular simulations. Acc Chem Res.

[237] Singharoy A., Teo I., McGreevy R., Stone J.E., Zhao J., Schulten K. (2016). Molecular dynamics-based refinement and validation for sub-5 Å cryo-electron microscopy maps. eLife.

[238] Mirjalili V., Noyes K., Feig M. (2014). Physics-based protein structure refinement through multiple molecular dynamics trajectories and structure averaging. Proteins Struct Funct Genet.

[239] Gniewek P., Kolinski A., Jernigan R.L., Kloczkowski A. (2012). Elastic network normal modes provide a basis for protein structure refinement. J Chem Phys.

[240] Schneider J., Korshunova K., SiChaib Z., Giorgetti A., Alfonso-Prieto M., Carloni P. (2020). Ligand pose predictions for human G Protein-Coupled Receptors: insights from the Amber-based hybrid molecular mechanics/coarse-grained approach. J Chem Inf Model.

[241] Wang A., Zhang Y., Chu H., Liao C., Zhang Z., Li G. (2020). Higher accuracy achieved for protein-ligand binding pose prediction by Elastic Network Model-based ensemble docking. J Chem Inf Model.

[242] Cavasotto C.N., Baron R. (2012). Computational drug discovery and design, methods in molecular biology.

[243] Evangelista Falcon W., Ellingson S.R., Smith J.C., Baudry J. (2019). Ensemble docking in drug discovery: how many protein configurations from molecular dynamics simulations are needed to reproduce known ligand binding?. J Phys Chem B.

[244] Stansfeld P.J., Sansom M.S.P. (2011). From coarse grained to atomistic: a serial multiscale approach to membrane protein simulations. J Chem Theory Comput.

[245] Noé F., Tkatchenko A., Müller K.-R., Clementi C. (2020). Machine learning for molecular simulation. Annu Rev Phys Chem.

[246] Noé F., De Fabritiis G., Clementi C. (2020). Machine learning for protein folding and dynamics. Curr Opin Struct Biol.

[247] Jin Y., Johannissen L.O., Hay S. (2021). Predicting new protein conformations from molecular dynamics simulation conformational landscapes and machine learning. Proteins Struct Funct Bioinform.

[248] Karamzadeh R., Karimi-Jafari M.H., Sharifi-Zarchi A., Chitsaz H., Salekdeh G.H., Moosavi-Movahedi A.A. (2017). Machine learning and network analysis of molecular dynamics trajectories reveal two chains of red/ox-specific residue interactions in human protein Disulfide Isomerase. Sci Rep.

[249] Spiwok V., Kr^íž P. (2020). Time-lagged t-Distributed Stochastic Neighbor Embedding (t-SNE) of molecular simulation trajectories. Front Mol Biosci.

[250] Wang D.D., Ou-Yang L., Xie H., Zhu M., Yan H. (2020). Predicting the impacts of mutations on protein-ligand binding affinity based on molecular dynamics simulations and machine learning methods. Comput Struct Biotechnol J.

[251] Marchetti F., Moroni E., Pandini A., Colombo G. (2021). Machine learning prediction of allosteric drug activity from molecular dynamics. J Phys Chem Lett.

[252] Glazer D.S., Radmer R.J., Altman R.B. (2009). Improving structure-based function prediction using molecular dynamics. Structure.

[253] C. Outeiral, D.A. Nissley, C.M. Deane, Current protein structure predictors do not produce meaningful folding pathways, bioRxiv Preprint (Sep. 2021). 10.1101/2021.09.20.461137.

[254] Hochuli J., Helbling A., Skaist T., Ragoza M., Koes D.R. (2018). Visualizing convolutional neural network protein-ligand scoring. J Mol Graph Model.

[255] Kim E., Goren A., Ast G. (2008). Alternative splicing: current perspectives. BioEssays.

[256] Owji H., Nezafat N., Negahdaripour M., Hajiebrahimi A., Ghasemi Y. (2018). A comprehensive review of signal peptides: structure, roles, and applications. Eur J Cell Biol.

[257] Ribeiro A.J.M., Das S., Dawson N., Zaru R., Orchard S., Thornton J.M., Orengo C., Zeqiraj E., Murphy J.M., Eyers P.A. (2019). Emerging concepts in pseudoenzyme classification, evolution, and signaling. Sci Signal.

[258] Smith L.M., Kelleher N.L. (2018). Proteoforms as the next proteomics currency. Science.

[259] Camacho D.M., Collins K.M., Powers R.K., Costello J.C., Collins J.J. (2018). Next-generation machine learning for biological networks. Cell.

[260] Fuentealba M., Dönertas H.M., Williams R., Labbadia J., Thornton J.M., Partridge L. (2019). Using the drug-protein interactome to identify anti-ageing compounds for humans. PLoS Comput Biol.

[261] Murray D., Petrey D., Honig B. (2021). Integrating 3D structural information into systems biology. J Biol Chem.

[262] Aloy P., Russell R.B. (2006). Structural systems biology: modelling protein interactions. Nat Rev Mol Cell Biol.

